# Proteasome Inhibition Augments Cigarette Smoke-Induced GM-CSF Expression in Trophoblast Cells via the Epidermal Growth Factor Receptor

**DOI:** 10.1371/journal.pone.0043042

**Published:** 2012-08-17

**Authors:** Ya-Yuan Fu, Jennifer C. Nergard, Nicole K. Barnette, Yan-Ling Wang, Karl X. Chai, Li-Mei Chen

**Affiliations:** 1 State Key Laboratory of Reproductive Biology, Institute of Zoology, Chinese Academy of Sciences, Beijing, People's Republic of China; 2 Burnett School of Biomedical Sciences, University of Central Florida College of Medicine, Orlando, Florida, United States of America; Institute of Zoology, Chinese Academy of Sciences, China

## Abstract

Maternal cigarette smoking has adverse effects on pregnancy outcomes. The granulocyte-macrophage colony-stimulating factor (GM-CSF) is an essential cytokine for a normal pregnancy. We investigated the impact of cigarette smoke extract (CSE) on GM-CSF expression in human cytotrophoblast cells and suggested a cellular mechanism underlying the CSE-induced GM-CSF expression. An immortalized normal human trophoblast cell line (B6Tert-1) was treated with CSE. The viability and proliferation of the CSE-treated B6Tert-1 cells were evaluated, and the expression of GM-CSF in these cells was quantified at the mRNA and the protein levels by means of reverse-transcription and quantitative polymerase chain reaction (RT-qPCR); and enzyme-linked immunosorbent assay (ELISA), respectively. Human trophoblast cells treated with CSE had an increased expression of GM-CSF at both the mRNA and the protein levels. The CSE-induced GM-CSF expression was synergistically enhanced by the addition of the proteasome inhibitor MG-132, but inhibited by AG-1478, an inhibitor of the epidermal growth factor receptor (EGFR) kinase. Furthermore, CSE treatment increased the phosphorylation of the extracellular-signal regulated kinases (ERK1/2) in the trophoblast cells. The expression of other growth factors such as heparin-binding epidermal growth factor-like growth factor (HB-EGF) and vascular endothelial growth factor (VEGF) was also evaluated. Our data suggested that cigarette smoking and proteasome inhibition synergistically up-regulate GM-CSF cytokine expression by activating the EGFR signaling pathway.

## Introduction

Maternal cigarette smoking has negative impacts on all aspects of human reproduction, causing impaired fertility, increased risk of pregnancy complications and poor pregnancy outcomes such as fetal growth restriction, premature delivery, fetal and infant death, and developmental problems with the newborn [1]–[5]. Paradoxically, maternal cigarette smoking is also associated with a reduced risk of preeclampsia by up to 50% with a dose-response pattern [6]–[9]. Smokers with preeclampsia, however, have very high risks of even worse outcomes than nonsmokers [10]. Preeclampsia affects about 5–10% of all pregnancies and is a leading cause of maternal and fetal/neonatal morbidity and mortality worldwide [11]. Smoking may have effects on angiogenesis of placenta arteries, endothelial function and the immune system, but the underlying mechanisms are not fully understood.

The granulocyte-macrophage colony-stimulating factor (GM-CSF) is a hematopoietic cytokine which plays an important role in the proliferation, differentiation and function of myeloid cells [12], and is an important regulator of the host defense and response to external insult and injury [13]. Other studies suggested that GM-CSF also plays a key role in embryo development by regulating the cell number and viability of mouse and human blastocysts [14]. Aberrant GM-CSF expression will have impacts on embryo implantation as well as on fetal and placental development. Mice lacking the GM-CSF gene had impaired fertility, fetal growth retardation, and fetal loss in late gestation [15], [16]. Administration of GM-CSF exogenously could protect against embryo loss and enhance fetal growth [17]–[19].

Throughout pregnancy, a high level of GM-CSF expression can be observed at the feto-maternal interface, as well as in the invading cytotrophoblast cells [20]. GM-CSF could provide the necessary signals for trophoblast differentiation and function. The levels of GM-CSF in the peripheral blood of women with normal pregnancy or preeclampsia have been evaluated, but the results were controversial. Hayashi and colleagues [21] reported that the level of GM-CSF is significantly higher in the peripheral blood and the placenta of preeclamptic women than in the specimens of women with normal pregnancy. In other reports, however, no significant differences were found in the plasma levels of GM-CSF between preeclamptic women and those with normal pregnancy [22]–[25].

The regulation of GM-CSF expression in human trophoblast cells under the influence of cigarette smoking has not been well studied. The aim of this study was to investigate the effect of cigarette smoke extract (CSE) on GM-CSF expression in normal human trophoblast cells. We used an immortalized human normal cytotrophoblast cell line to investigate GM-CSF expression in the absence or presence of CSE in the culture medium. We demonstrated that proteasome inhibition leads to a significant enhancement of CSE-induced GM-CSF expression via the EGFR signaling pathway. The up-regulated expression of GM-CSF in the trophoblasts after CSE exposure could play an important role in maintaining trophoblast integrity to increase the chance of survival.

## Results

### Effects of cigarette smoke extract (CSE) on B6Tert-1 trophoblast cell viability and proliferation

**Figure 1:**
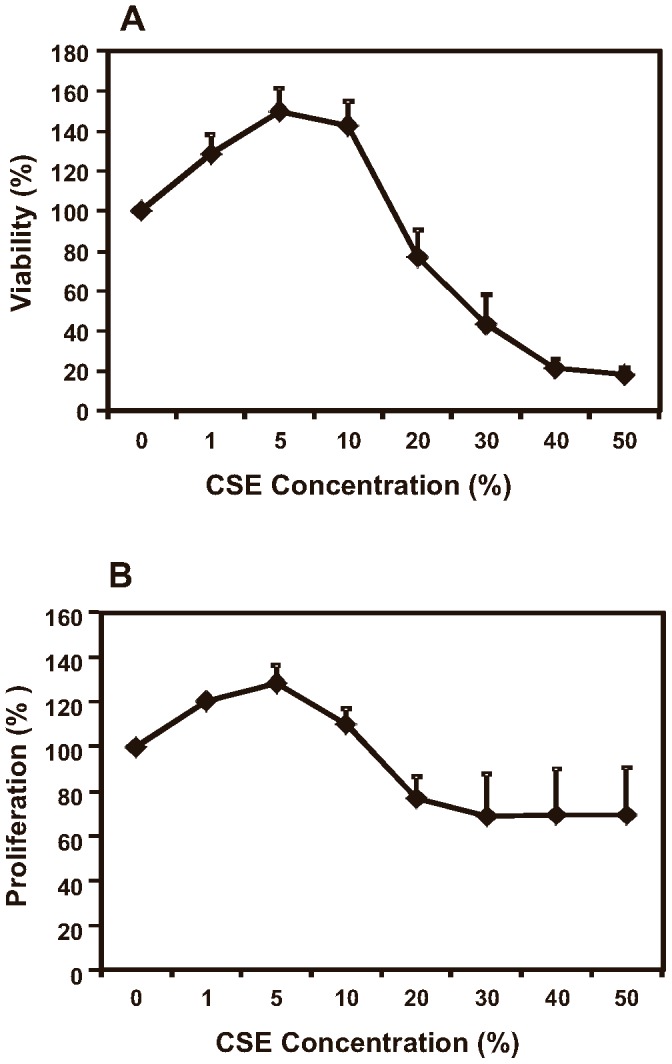
Viability and proliferation assays. B6Tert-1 cells (1×10^4^) were seeded in a 96-well plate in triplicates and grown overnight. Cigarette smoke extract (CSE) was added in FD medium at different final concentrations as indicated, and the cells were incubated for another 24 h. The viability and proliferation rate were monitored as described in Materials and Methods. The data are expressed as the percentage of CSE-treated/untreated, and represent the mean ± SEM. The experiment was repeated for three times.

The viability of the B6Tert-1 cells was increased by as much as 50% when cultured in medium containing 1% to 10% CSE (Figure 1A, *p*<0.05). The proliferation rate was increased by up to 29% when CSE at 1% to 5% was present in the medium (Figure 1B, *p*<0.05). As a result of the toxic effect of CSE at the higher concentrations (>20%) in the culture medium, the B6Tert-1 cells had a reduced proliferation rate, at 70% of that of the untreated cells; and a very low viability, at 20% to 40% of that of the untreated cells. CSE at a final concentration of 10% slightly increased B6Tert-1 cells' proliferation rate, by 10%, but not reaching statistical significance (*p*>0.05) compared to that of the untreated cells; while the 10% CSE in the medium increased the cell viability, by 43% (*p*<0.05). In the following experiments, a final CSE concentration of 10% was used to ensure that the viability and proliferation of the cells were not compromised by the presence of CSE.

### GM-CSF expression in B6Tert-1 cells under CSE exposure

**Figure 2:**
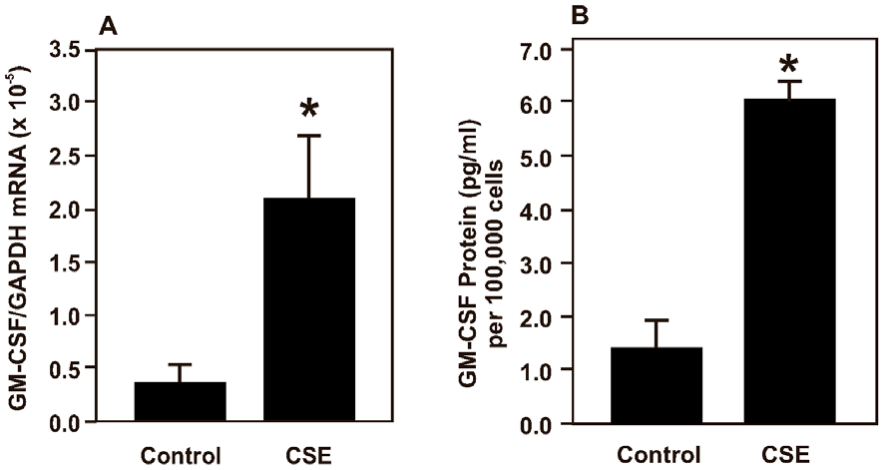
Cigarette smoke extract increases GM-CSF expression in B6Tert-1 cells. (A) Bar graph of real-time RT-qPCR data of GM-CSF mRNA expression in B6Tert-1 cells treated with 10% CSE in growth medium for 2 days. The relative GM-CSF mRNA expression level was determined against the glyceraldehyde-3-phosphate dehydrogenase (GAPDH) mRNA level. Data are mean ± SEM. The asterisk (*) indicates a statistically significant difference (*p*<0.05), when compared with the control (untreated) cells. (B) Bar graph of GM-CSF ELISA data of the secreted GM-CSF in B6Tert-1 conditioned medium. The medium was collected after 2 days of exposure to 10% CSE. The asterisk (*) indicates a statistically significant difference (*p*<0.05), when compared with the control (untreated) cells. Data are mean ± SEM.

CSE in the culture medium at a final concentration of 10% increased the GM-CSF expression in the B6Tert-1 cells at the mRNA level as measured by reverse-transcription and quantitative polymerase chain reaction (RT-qPCR) (Figure 2A). The GM-CSF mRNA expression increase was accompanied by an increased secretion of the GM-CSF protein in the culture medium (Figure 2B). We observed an up-regulation of GM-CSF mRNA expression to 5.7-fold, while the secretion of GM-CSF protein in the conditioned medium was increased to 4.3-fold.

### Proteasome inhibition and cellular distribution of NF-κB p65 subunit in B6Tert-1 cells under CSE exposure

**Figure 3:**
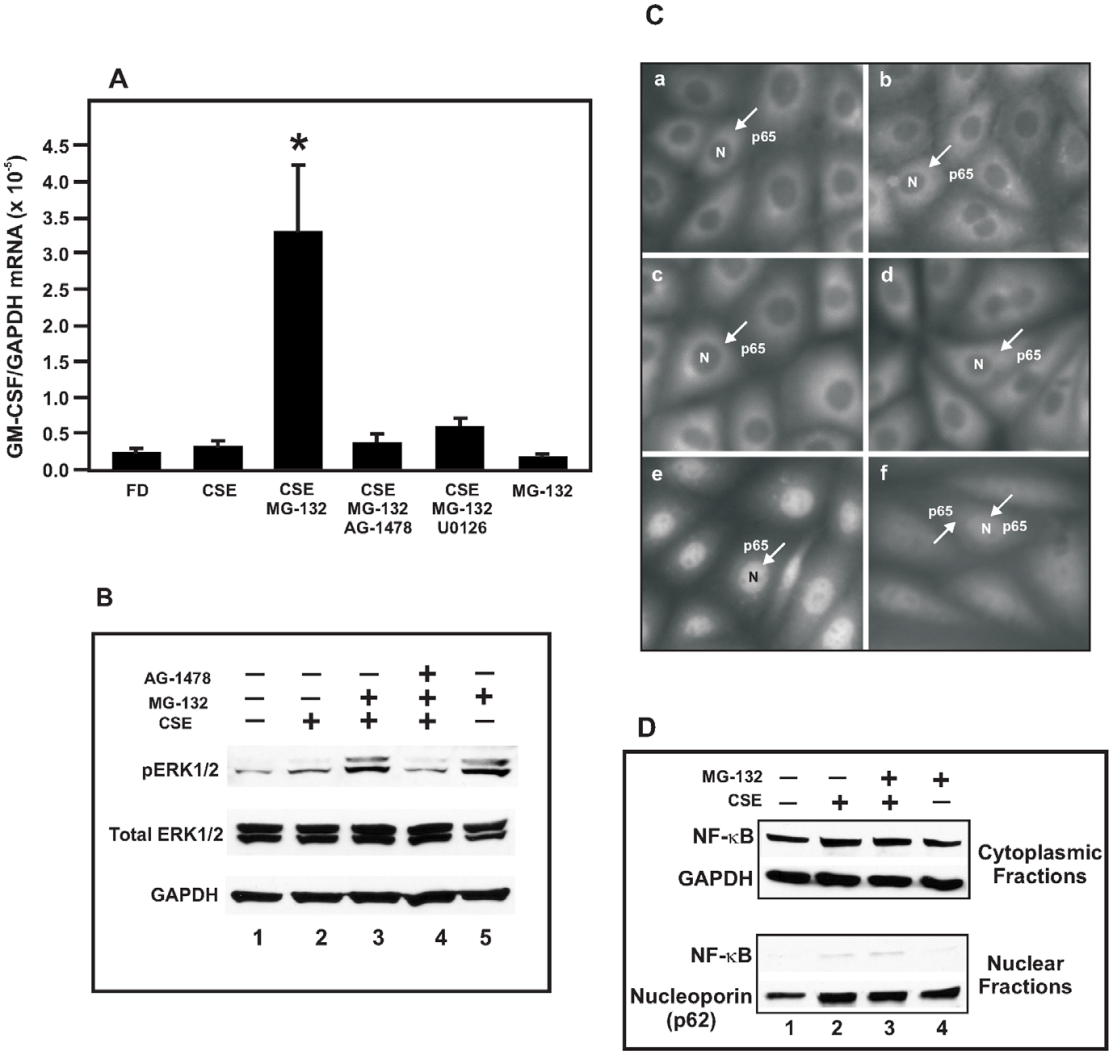
Effects of inhibitors on CSE-induced GM-CSF mRNA expression. (A) Changes of GM-CSF mRNA expression level in B6Tert-1 cells treated with different agents in FD medium. CSE: 10% cigarette smoke extract; MG-132: proteasome inhibitor at 5 µM; AG-1478: EGFR kinase inhibitor at 5 µM; U0126: MEK inhibitor at 5 µM. Cells were pre-treated with inhibitor(s) for 30 min, and then with 10% CSE for another 5 h. DMSO was used as a vehicle control. The asterisk (*) indicates a statistically significant difference (*p*<0.05) when compared with CSE-treated cells. (B) Western blot analysis of the phosphorylation state of ERK1/2 in B6Tert-1 cells treated with 10% CSE without or with inhibitors (5 µM each) as indicated for 30 min. Total ERK1/2 and GAPDH were used as the total protein and loading controls. Lane 1: FD (no treatment); lane 2: 10% CSE; lane 3: 10% CSE/MG-132; lane 4: 10% CSE/MG-132 and AG-1478; lane 5: MG-132 alone. The image represents one of three independently performed experiments. (C) Immunofluorescent staining showing the cellular distribution of NF-κB p65 subunit in B6Tert-1 cells under different treatment conditions. a: FD alone; b: 10% CSE; c: 10% CSE/MG-132; d: MG-132 alone; e: TNF-α: 50 ng/ml; and f: TNF-α/MG-132. “N” indicates the nucleus and the arrow indicates the NF-κB p65 subunit staining. Magnification: 20×10. (D) Western blot analysis of the distribution of NF-κB p65 subunit in B6Tert-1 cells under different treatment conditions. Cytoplasmic proteins were blotted with antibodies against NF-κB and GAPDH while nuclear proteins were blotted with antibodies against NF-κB and nucleoporin p62. Lanes 1: FD (no treatment); lanes 2: 10% CSE; lanes 3: 10% CSE/MG-132; and lanes 4: MG-132 alone. The image represents one of three independently performed experiments.

Previous studies have shown that NF-κB is a key transcriptional regulator of GM-CSF gene expression [26]. We investigated if this pathway might be involved in the CSE-induced GM-CSF transcription up-regulation. The B6Tert-1 cells were pre-treated with the proteasome inhibitor MG-132 at 5 µM for 30 min before exposure to 10% CSE for another 5 h. Due to the deleterious consequences of long-term proteasome inhibition by MG-132 on B6Tert-1 cell viability (data not shown), we treated the B6Tert-1 cells for 5 h with CSE in the presence of MG-132 for the evaluation of GM-CSF mRNA expression changes. Proteasome inhibition is expected to inactivate the NF-κB pathway by reducing the degradation of the IκB inhibitor molecules, thus preventing the translocation of the NF-κB transcription factor from the cytosol to the nucleus and preventing GM-CSF expression up-regulation. Unexpectedly, in the presence of the proteasome inhibitor, the CSE-induced GM-CSF expression was further up-regulated to ∼10-fold as compared to the GM-CSF expression level in cells treated with 10% CSE alone (Figure 3A). Of note, the cells treated with 10% CSE for 5 h (Figure 3A) had a less amount of GM-CSF mRNA as compared with those treated for 2 days (Figure 2A). In a western blot analysis, we observed an increased quantity of phosphorylated ERK1/2 (pERK1/2, Figure 3B, lanes 2 & 3), a signaling molecule downstream of EGFR in the CSE-treated or the CSE/MG-132-treated B6Tert-1 cells. The increase of pERK1/2 by the CSE/MG-132 treatment was blocked by the addition of AG-1478 (Figure 3B, lane 4). The addition of only MG-132 to the B6Tert-1 cells did not increase the expression of GM-CSF mRNA (Figure 3A), but increased ERK1/2 phosphorylation (Figure 3B, lane 5).

We further examined whether the transcription factor NF-κB was translocated into the nucleus after the CSE treatment by means of cellular immuno-fluorescent staining (Figure 3C) and nuclear protein western blotting (Figure 3D). As shown in Figure 3C, there was no apparent difference in the distribution of the NF-κB p65 subunit shown by the punctate staining surrounding the dark nuclear region (“N”) when cells were treated with the following agents: no-treatment (a), CSE alone (b), CSE/MG-132 (c), and MG-132 alone (d). TNF-α (tumor necrosis factor-alpha) was used as a control cytokine to show the translocation of NF-κB p65 subunit from the cytosol to the nucleus with the intense nuclear staining (e), indicating activation of the NF-κB pathway in the B6Tert-1 cells in response to a known NF-κB-activating cytokine [27]. The TNF-α-induced NF-κB translocation could be blocked by the addition of MG-132 (f). By means of western blot analysis, we showed that the majority of the NF-κB p65 subunit was detected in the cytoplasm in the B6Tert-1 cells treated with CSE, MG-132, or both (Figure 3D). Again, there was no apparent difference in the distribution of the NF-κB p65 subunit in the nucleus before and after the CSE treatment without or with proteasome inhibition. GAPDH and nucleoporin p62 were used as loading and fractionation controls in these western blot analyses. We also performed CSE treatment on the B6Tert-1 cells for 1 h, 3 h and 5 h (data not shown), the results of NF-κB immunostaining and western blotting were similar to those obtained from the cells treated for 30 min (shown in Figure 3C and 3D). These results indicated that the GM-CSF gene expression regulation by CSE in the trophoblast cells may involve a proteasome inhibition dependent but NF-κB independent mechanism.

### Up-regulation of GM-CSF expression in B6Tert-1 cells by CSE involves EGF/EGFR signaling

**Figure 4:**
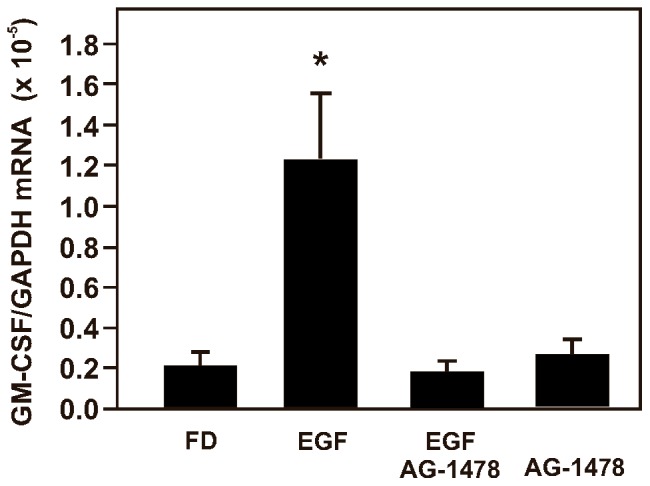
EGF up-regulates GM-CSF mRNA expression in B6Tert-1 cells. (A) Bar graph of real-time RT-qPCR data of GM-CSF mRNA expression in B6Tert-1 cells treated with EGF. FD: untreated; EGF: 50 ng/ml; AG-1478: 5 µM. Cells were pre-treated with AG-1478 for 30 min and then with 10% CSE for another 5 h. The asterisk (*) indicates a statistically significant difference (*p*<0.05) when compared with the untreated (FD) cells.

Next, we investigated if EGFR activation affected GM-CSF expression. We showed in Figure 3A that the EGFR kinase inhibitor AG-1478 blocked the CSE/MG-132-induced up-regulation of GM-CSF expression. Furthermore, an inhibitor of MEK1/2 (U0126) can also block the GM-CSF expression up-regulation induced by CSE/MG-132. The involvement of EGF/EGFR signaling pathway in the up-regulation of GM-CSF expression was further supported by the results of treating the B6Tert-1 cells with EGF (Figure 4). The addition of EGF to the culture medium (FD) increased GM-CSF mRNA expression to 5.7-fold in 5 h of treatment. AG-1478 alone did not affect the GM-CSF mRNA expression, but blocked the induction of GM-CSF mRNA expression under EGF treatment.

### GM-CSF or EGF increases the viability and proliferation of B6Tert-1 cells

**Figure 5:**
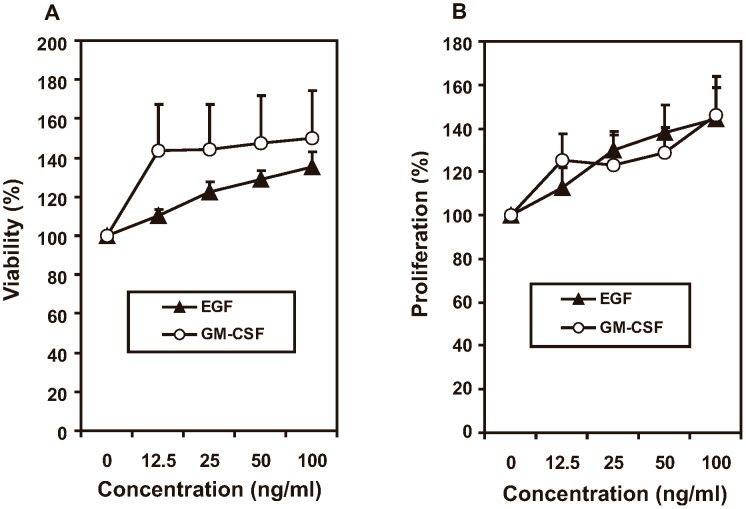
GM-CSF and EGF increase viability and proliferation of B6Tert-1 cells. B6Tert-1 cells were seeded in 96-well plates and treated with GM-CSF or EGF at different concentrations as indicated in FD medium for 24 h. The data are expressed as the percentage of treated/untreated. Each data point represents mean ± SEM (n = 7).

The responses of the B6Tert-1 trophoblast cells to GM-CSF or EGF, manifested as changes in cell viability and proliferation were shown in Figure 5. Cell viability and proliferation of the B6Tert-1 trophoblast cells were both increased in a dose-dependent manner (*p*<0.05) when GM-CSF or EGF was added to the culture medium. The cell viability was increased by up to 50% or 36% by GM-CSF (100 ng/ml) or EGF (100 ng/ml), respectively, while the proliferation rate was increased by up to 46% or 45%, respectively, by these two agents. The results suggested that both GM-CSF and EGF are important regulators of trophoblast cell function.

### VEGF and HB-EGF expression in B6Tert-1 cells under CSE exposure

**Figure 6:**
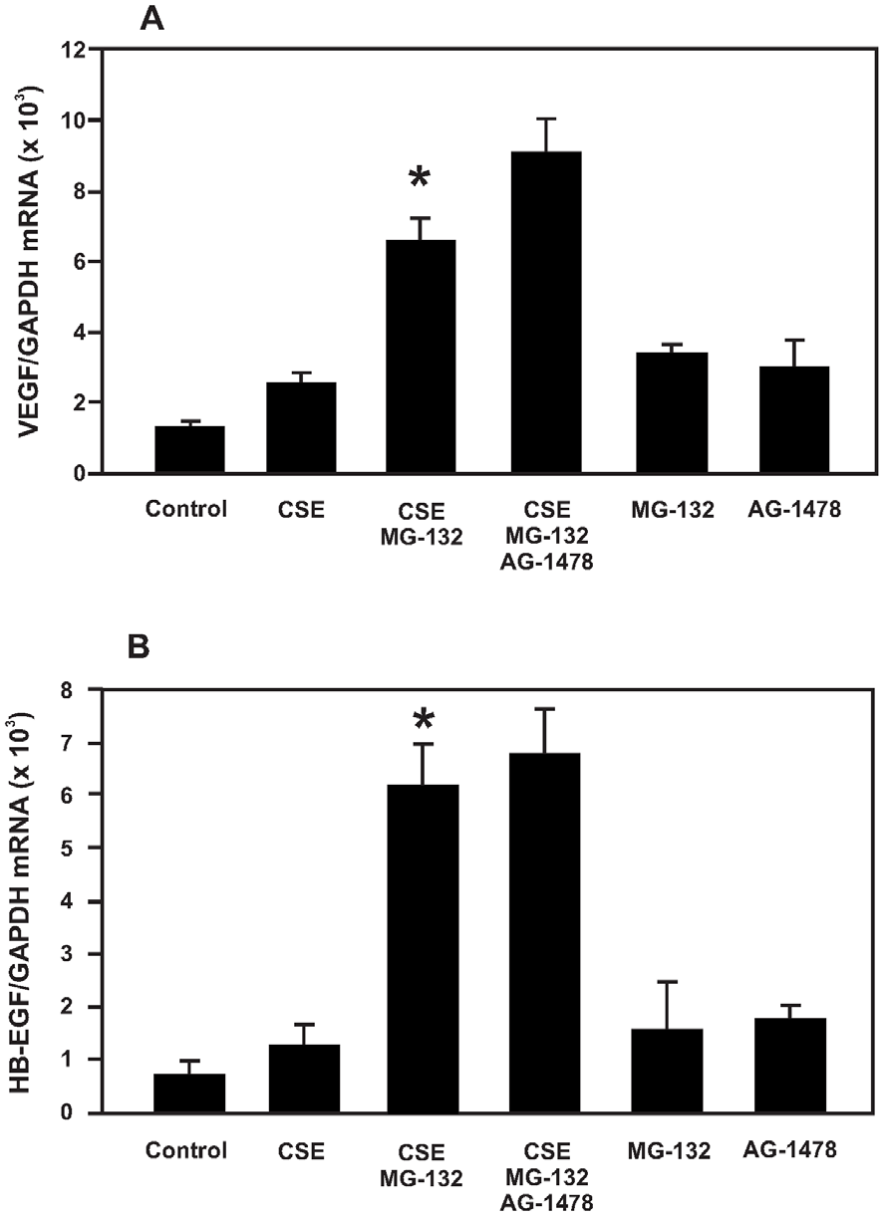
Effects of CSE and proteasome inhibition on VEGF and HB-EGF mRNA expression. Cells were pre-treated with inhibitor(s) for 30 min, and then with 10% CSE for another 5 h. DMSO was used as a vehicle control. A: VEGF mRNA expression levels in B6Tert-1 cells treated with different agents in FD medium. B: HB-EGF mRNA expression levels in B6Tert-1 cells treated with different agents in FD medium. CSE: 10% cigarette smoke extract; MG-132: proteasome inhibitor at 5 µM; AG-1478: EGFR kinase inhibitor at 5 µM. The asterisk (*) indicates a statistically significant difference (*p*<0.05) when compared with CSE-treated cells.

We examined the response of two other important mitogens in B6Tert-1 cells upon CSE and/or MG-132 treatment. As shown in Figure 6, the expression of VEGF and HB-EGF was increased in B6Tert-1 cells at the mRNA level under CSE exposure and was further increased when MG-132 was present during the CSE treatment. However, this up-regulation was not blocked by the EGFR inhibitor AG-1478. These results showed that the synergistic up-regulation of VEGF and HB-EGF expression by CSE and MG-132 was not by way of activating EGFR as was the case for the GM-CSF expression up-regulation by CSE and MG-132. The addition of only MG-132 to the B6Tert-1 cells also increased the expression of VEGF and HB-EGF mRNAs, but not to the extent observed when both CSE and MG-132 were present in FD medium.

## Discussion

Cigarette smoke contains about 4,000 toxic compounds [28]. It is difficult to single out which chemical compound is responsible for the adverse effects on human health since a smoker is not smoking any single compound. It is the combined actions of all these damaging compounds modifying the cellular signaling pathways over time that define the overall impact of cigarette smoke on the human body. With this consideration, we chose to use the whole cigarette smoke extract (CSE) for the present study. The dose of soluble CSE described here is comparable to those in previously published studies [29], [30] and is pharmacologically relevant when compared with the plasma nicotine concentrations of smokers [31], [32].

The regulation of GM-CSF expression in human trophoblast cells under the influence of cigarette smoking has not been well studied previously. In this report, we demonstrated that CSE increased GM-CSF expression and this induction effect is mediated by a cellular signaling pathway involving EGFR activation and proteasome inhibition. The transcription factor NF-κB plays important roles in pro-inflammatory cytokine mRNA expression and GM-CSF is a target gene of this expression regulation [26]. Our results, however, suggested that GM-CSF mRNA expression can be up-regulated via EGFR activation and proteasome inhibition in trophoblast B6Tert-1 cells under the exposure of cigarette smoke by an NF-κB independent mechanism. In the cells treated with CSE under our experimental conditions (Figure 3C–D), there was no apparent translocation of the NF-κB p65 subunit from the cytosol to the nucleus, a process necessary for NF-κB-mediated transcription activation. On the other hand, in the cells treated with the known NF-κB-activating cytokine TNF-α, the NF-κB p65 subunit was translocated into the nucleus, and the translocation could be blocked by the proteasome inhibitor MG-132; indicating that the B6Tert-1 cells can respond to cytokine stimulation with a proper NF-κB activation [33]. The fact that an NF-κB activation-inhibiting proteasome inhibitor (MG-132) further enhanced the CSE-induced GM-CSF mRNA expression (Figure 3A) suggests that the CSE-mediated GM-CSF up-regulation in the trophoblast cells is the consequence of impaired proteasome function. Similarly, nicotine-induced inhibition of proteasomal chymotrypsin-like activity was observed in the mouse brain in animals injected with nicotine [34]. Proteasome inhibition has recently been shown to increase NF-κB-mediated transcription instead of inhibiting it in endometrial cancer cells, with an increase in nuclear NF-κB and NF-κB/target gene promoter complex formation [35]. But in our study, the CSE-induced and MG-132-enhanced GM-CSF expression may not be attributable to NF-κB activation, or retention of NF-κB on the promoter following activation and translocation as previously reported [36].

The CSE/MG-132-induced GM-CSF mRNA expression was prevented by AG-1478, a highly selective inhibitor of the EGFR kinase (Figure 3A), suggesting that the GM-CSF mRNA expression up-regulation was regulated via activation of EGFR signaling. Indeed, treating B6Tert-1 trophoblast cells with EGF resulted in an increased GM-CSF mRNA expression (Figure 4). Our results were consistent with the previous findings that EGFR can be aberrantly activated by cigarette smoke which also impairs EGFR degradation, leading to downstream ERK1/2 phosphorylation in human airway epithelial cells [37], and that EGFR activation is associated with an enhanced expression of GM-CSF in keratinocyte [38]. Our data delineate a possible mechanism underlying the CSE/MG-132-induced GM-CSF up-regulation: that the increased ERK1/2 phosphorylation activates the GM-CSF transcription. Indeed, treating B6Tert-1 cells with U0126, a MEK1/2 inhibitor, inhibited CSE/MG-132-induced GM-CSE up-regulation (Figure 3A). As shown in Figure 3B, the addition of only MG-132 increased ERK1/2 phosphorylation in the B6Tert-1 cells; a phenomenon well documented in previous publications [39]–[41]. The increased ERK1/2 phosphorylation by MG-132 would exert different functional outputs depending on the cell state dictated by the presence of other stimuli. In our experiments, CSE in addition to MG-132 synergistically increased the expression of GM-CSF.

Cigarette smoking during pregnancy is known to increase the risks of a number of adverse outcomes, such as fetal growth restriction and preterm birth. Paradoxically, smoking reduces the risk of preeclampsia. We have shown in this study that cigarette smoke extract in the culture medium of a trophoblast cell line, mimicking systemic cigarette smoke exposure, induces GM-CSF expression. The GM-CSF up-regulation in response to cigarette smoking may be a compensatory mechanism for repairing the pathological damages induced by cigarette smoking in the trophoblast based-placental structures, working in favor of sustaining the pregnancy. As presented in Figure 5, treating B6Tert-1 cells with exogenous GM-CSF increased trophoblast cell viability and proliferation. Our results corroborated previous reports that GM-CSF and its receptors expressed by trophoblast cells provide onsite actions of regulating trophoblast cell proliferation, invasion and differentiation [42]. The increased expression of GM-CSF upon CSE treatment in B6Tert-1 cells may explain, in part, that low doses of CSE increased the cell viability and proliferation (Figure 1). In addition, CSE and/or MG-132 treatment increased other growth promoting factors in B6Tert-1 cells, i.e., VEGF or HB-EGF, but via different mechanisms.

**Figure 7:**
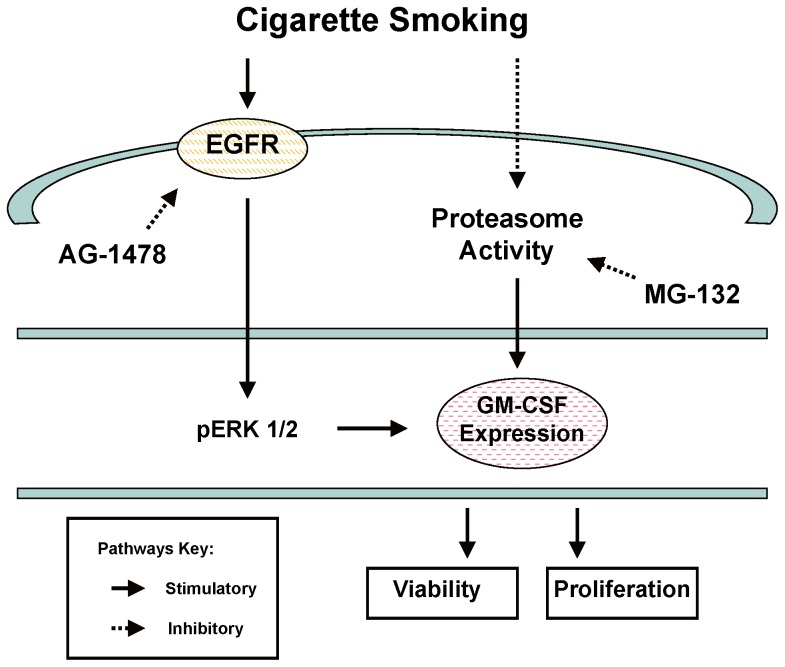
Cigarette smoke activates different signaling pathways regulating trophoblast cell viability and proliferation via GM-CSF expression.

Although smoking is associated with a reduced incidence of preeclampsia, the net effect of smoking in regard to pregnancy outcomes is undoubtedly unfavorable. But the important lesson from this study is that we have identified the cytokine GM-CSF as being a possible mediator of such a dubious benefit of maternal cigarette smoking. The most significant and novel finding of this study is that the combination of CSE exposure and proteasome inhibition increased extracellular signal-regulated kinases (ERK1/2) phosphorylation, via activating the EGFR, to serve as a possible mechanism underlying the CSE/proteasome inhibition-induced GM-CSF up-regulation. The up-regulated expression of GM-CSF in the trophoblasts after cigarette smoke exposure could play an important role in maintaining trophoblast integrity to increase the survival of cells (Figure 7). Previous studies have demonstrated that proteasome activity is impaired in placenta in women with preeclampsia [43], [44]. Our finding in this study will allow us to dissect the molecular and cellular events associated with the reduced-risk of preeclampsia in smokers, and take full advantage of the benefits endowed by GM-CSF to pregnant women highly prone to preeclampsia, without having to derive these benefits from cigarette smoking.

## Materials and Methods

### Materials

All tissue culture reagents, cytokines GM-CSF and TNF-α, and the Trizol reagent were purchased from Invitrogen (Carlsbad, CA, USA) unless specified otherwise. Inhibitors MG-132, AG-1478 and U0126 were purchased from CalBiochem (San Diego, CA, USA). Other chemicals were purchased from Sigma-Aldrich (St. Louis, MO, USA).

### Cell culture

An immortalized normal human cytotrophoblast cell line, B6Tert-1, was established, characterized, and cultured as described previously [45]. All cell culture dishes were pre-coated with 75 µg/ml Cellmatrix Type I-A (Institute of Biochemistry, Osaka, Japan). The B6Tert-1 cells were cultured in F12/DMEM (1∶1) medium (FD) supplemented with 10 ng/ml EGF, 2 mM glutamine, 10 µg/ml insulin and 0.1%BSA; in a humidified incubator at 37°C with 5% CO_2_ and 95% air.

### Cigarette smoke extract preparation

Research-reference cigarettes were obtained from the Kentucky Tobacco Research and Development Center, University of Kentucky (Lexington, KY, USA). Cigarette smoke extract (CSE) was prepared as described previously with modifications [46]–[48]. Smoke from one research-grade filter-less cigarette, 1R3F containing 15 mg tar and 1.16 mg nicotine, was bubbled through 25 ml of FD medium via a 10-ml glass syringe at a rate of 5 min per cigarette. The CSE stock (considered as 100%) was filtered through a 0.22-µm filter and stored at −80°C. The CSE was diluted in FD medium immediately prior to use.

### Treatment of cells

Cells were seeded in 12-well culture plates at a density of 3×10^5^ cells per well and grown overnight. Inhibitors MG-132, AG-1478, or U0126 were added, when appropriate, in FD medium 30 min prior to CSE treatments. CSE was added to the cell culture at a final concentration of 10% in FD medium with the added inhibitor(s), and was incubated with cells for 5–6 hours. At the end of the treatment, cells were either lysed with the Trizol reagent for total cellular RNA isolation, or lysed with the RIPA buffer for total cellular protein extraction.

### Cell viability and proliferation assays

Cells were seeded into a 96-well plate at a density of 1×10^4^/well and cultured overnight. Cells were then treated with CSE at different concentrations (0%, 1%, 5%, 10%, 20%, 30%, 40%, and 50%) in a volume of 100 µl/well for 24 h in FD medium. EGF or GM-CSF was added, when appropriate, at different final concentrations. Cell viability was evaluated using the CellTiter-Blue® Cell Viability Assay Kit (Promega, Madison, WI, USA). Cell proliferation was evaluated using the CyQUANT® NF Cell Proliferation Assay Kit (Invitrogen).

### RNA isolation and real-time RT-qPCR

Total cellular RNA was isolated using the Trizol reagent according to the manufacturer's protocol. Reverse transcription of each sample was carried out using the iScript cDNA Synthesis Kit (Bio-Rad, Hercules, CA). Real-time qPCR was carried out on a Bio-Rad MyiQ system using the iQ SYBR Green Supermix reagents (Bio-Rad). The PCR program and methods of quantification were described previously [49]. PCR primers were designed with the aid of the Beacon Designer 7.0 software (PREMIER BioSoft International, Palo Alto, CA) and synthesized by Integrated DNA Technologies (Coralville, IA, USA). The primer sequences for human glyceraldehyde-3-phosphate dehydrogenase (GAPDH) and GM-CSF were described previously [49]. The primer sequences for HB-EGF are 5′- AAT CGC TTA TAT ACC TAT GAC-3′ (forward) and 5′- TAA CCT CCT CTC CTA TGG-3′ (reverse); those for VEGF are 5′- TTG CTG CTC TAC CTC CAC-3′ (forward) and 5′- CAC AAG ATG GCT TGA AGA TG-3′ (reverse). The relative mRNA expression of target genes in each RNA sample was calculated as copy numbers per GAPDH mRNA copy.

### Enzyme-linked immunosorbent assay (ELISA)

Cells were seeded in 12-well culture plates at a density of 1×10^5^ cells per well and grown overnight, and then treated with 10% CSE in fresh culture medium for another 48 h. The GM-CSF protein secreted in the culture medium was quantified using a Quantikine™ human GM-CSF immunoassay kit (R&D Systems, Minneapolis, MN, USA) as described previously [49]. The quantities of GM-CSF were expressed as pg/ml after normalizing with the cell number in the culture dishes.

### Immunofluorescent staining

Cells were seeded on 12-mm diameter glass coverslips (Thermo Fisher Scientific, Waltham, MA, USA) at 5×10^4^ cells per coverslip, grown overnight, and then treated with different agents in FD medium. After the treatments, cells were washed with cold PBS and fixed with 4% para-formaldehyde at 4°C for 15 min and then with cold acetone:alcohol (1∶1) for another 10 min. The cells were washed with PBS and then blocked with 1% BSA in PBS containing 0.1% Triton X-100 for 30 min. The cells were then incubated with the primary antibody against NF-κB p65 subunit at 1∶100 dilution (Santa Cruz Biotechnology, Santa Cruz, CA) in the blocking buffer for 1 h at the room temperature. A goat anti-mouse IgG conjugated with Cy2 in the blocking buffer was applied to the cells at 1∶200 dilution and incubated for 1 h (Jackson ImmunoResearch laboratories, Pennsylvania, PA, USA) following the primary antibody incubation and washing in PBS. After a final wash with PBS, the cells were mounted with Biomeda Gel/Mount™ (Thermo Fisher Scientific), viewed and photographed under a Nikon, Eclipse TE2000E microscope equipped with the NIS-Element Advanced Research software.

### Nuclear Protein Extraction

Cytoplasmic and nuclear proteins were extracted using a Nuclear Extract Kit (Active Motif, Carlsbad, CA) according to the manufacturer's instructions. Briefly, cells were seeded in 60-mm tissue culture dishes (Corning Incorporated, Corning, NY) at 2×10^6^ cells per dish, grown overnight, and then treated with the different agents in FD medium. Extracted proteins were quantified using a DC Protein Assay Kit (Bio-Rad) and subjected to electrophoresis followed by western blot analysis as described below.

### Western Blot Analysis

The procedures to evaluate protein expression changes in the B6Tert-1 cells treated with the different agents were carried out as described previously [50]. Briefly, cells were washed with PBS and lysed in RIPA buffer at 4°C for 30 min (RIPA with inhibitors: 20 mM Tris-HCl at pH 8.0, containing 150 mM NaCl, 1% NP-40, 0.5% sodium deoxycholate, and 0.1%SDS; and enzyme inhibitors: 1 µM PMSF, 2 µg/ml aprotinin, 2 µg/ml leupeptin, 2 µg/ml antipain, 50 µg/ml soybean trypsin inhibitor, 10 mM NaF, 1 mM Na_3_VO_4_). The supernatant was collected after centrifugation of the lysate at 10,000× *g* for 10 min. The protein concentrations were determined using a DC Protein Assay Kit. Forty-micrograms of the total protein from each sample were resolved on a 10% gel by SDS-PAGE and electro-transferred to a nitrocellulose membrane. The membranes were blocked with 5% non-fat milk in TBS-T (20 mM Tris-HCl at pH 7.4, containing 150 mM NaCl, 0.1% Tween-20), and incubated with the appropriate primary antibodies. The antibodies used were GAPDH (used at 1∶5,000 dilution), total ERK1/2 (1∶5,000), and NF-κB (1∶1,000) (Santa Cruz Biotechnology); phosphorylated ERK1/2 (1∶1,000; Cell Signaling Technology, Inc., Danvers, MA); and nucleoporin p62 (1∶2,000; Pharmingen, San Diego, CA). After incubation with the primary antibodies, the membranes were washed and incubated for 1 h with the appropriate secondary antibodies conjugated with the horseradish peroxidase (HRP) (1∶10,000, Promega). The membranes were then washed and subjected to enhanced-chemiluminescence reaction (ECL, Pierce Biotechnology, Inc., Rockford, IL, USA) before exposure to X-ray films.

### Statistical analysis

All experiments were performed at least three times. All data are expressed as means ± SEM. All data were analyzed using Student's *t*-Test. Differences were considered statistically significant, if *p*<0.05.
